# Resonance-enhanced multiphoton ionization time-of-flight mass spectrometry for direct analysis of liposome-encapsulated compounds

**DOI:** 10.1007/s44211-024-00683-8

**Published:** 2024-11-07

**Authors:** Keishi Yamashita, Kento Sakakibara, Yuta Kouyama, Hitomi Sugiyama, Tomohiro Ueyama, Koji Nishijima, Tomohiro Uchimura

**Affiliations:** 1 Department of Materials Science and Engineering, Graduate School of Engineering, University of Fukui, 3-9-1 Bunkyo, Fukui, 910-8507 Japan; 2 General Center for Perinatal, Maternal and Neonatal Medicine, Niigata University Medical and Dental Hospital, 1-754, Asahimachi-dori, Chuo-ku, Niigata, 951-8520 Japan

**Keywords:** REMPI–TOFMS, Liposome

## Abstract

The direct measurement of compounds encapsulated into liposomes without pretreatment allows verification of both the encapsulation efficiency and the release rate of liposomes in their original state. In the present study, the direct analysis of liposomes was conducted via resonance-enhanced multiphoton ionization time-of-flight mass spectrometry (REMPI–TOFMS). When analyte species (2-phenoxyethanol) encapsulated in liposomes were measured online, spike signals appeared in a time profile of the peak area for 2-phenoxyethanol, which suggested a dispersion of the compound in this sample. In addition, the spikes disappeared when the liposomes collapsed following the addition of a Triton X-100 aqueous solution. These results strongly suggest that the appearance of spikes arises from the compound encapsulated into the dispersed liposomes. REMPI–TOFMS has an inherent characteristic of superior selectivity, which suggests that this process would be useful for achieving a precise evaluation of the release properties of target compounds even in a liposome sample containing a large variety of components.

**Graphical abstract:**

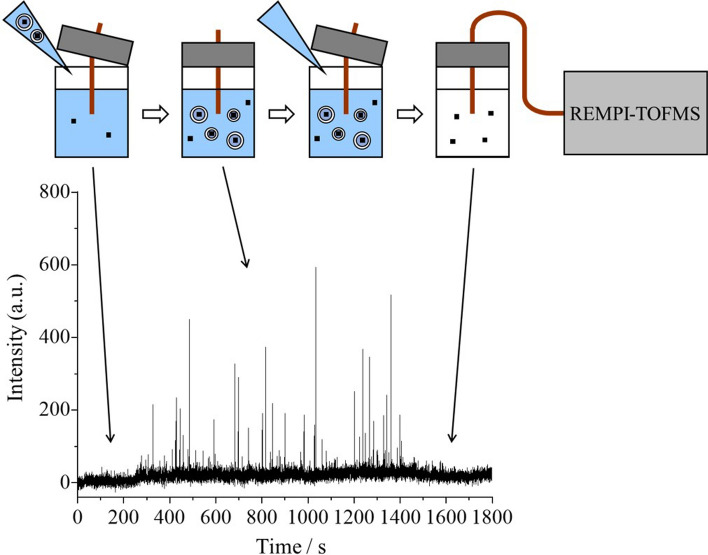

## Introduction

A liposome is a small sphere-shaped vesicle with a phospholipid bilayer. Since discovery by Bangham [1], liposomes have been investigated in areas such as biology and medical science due to a level of biocompatibility that allows the formulation of encapsulated medicines [2] and use as radioactive tracers for tumor imaging [3]. Many reports on detection of the constituents encapsulated into liposomes have focused on encapsulation efficiency and the release rate. Following ultracentrifugation, Katayama et al. used a spectrophotometer to measure the amount of a target drug in the supernatant, which allowed them to deduce the encapsulation efficiency [4]. Hummon et al. used matrix-assisted laser desorption/ionization imaging mass spectrometry (MALDI–IMS) and fluorescence microscopy to visualize liposomal drug penetration into spheroids [5]. Harris et al. employed confocal Raman microscopy to study the temperature-dependent release of encapsulated molecules [6]. There is a possibility that liposomes collapse during pretreatment processes such as centrifugation and drying. Therefore, to verify the release behavior of components encapsulated in liposomes, an analytical method that could achieve real-time monitoring without the need of pretreatments is quite useful.

Resonance-enhanced multiphoton ionization time-of-flight mass spectrometry (REMPI–TOFMS) is a highly selective analytical method [7–10]. REMPI is one of the ionization methods for mass spectrometry, and in the case of REMPI using ultraviolet laser pulses, target molecules with the same absorption wavelength as that of the laser wavelength are sensitively ionized. In many cases, the detectable components are aromatic compounds, and even in a mixture sample, such components could be selectively ionized. In addition, TOFMS is a non-scanning type of mass spectrometer that allows the simultaneous detection of all ions to be induced. We have applied this method to the online measurement of an emulsion without pretreatment [11]; an emulsion is a mixture of two or more immiscible liquids in which one is present as small droplets distributed throughout the other. When an oil-in-water (O/W) emulsion is measured online via REMPI–TOFMS, detectable aromatic compounds are usually employed either as an oil component or as a tracer component that exists mainly in an oil phase. In either case, from a series of obtained mass spectra, a time profile of a peak area arising from a detectable oil component is constructed. In such a profile positive spikes often are detected; the occurrence of these signals indicates that an oil phase is dispersed as small droplets with detectable sizes [12–14]. In addition, such components are seldom present in a water phase, which is recognized as a base signal in the time profile. Formerly, we applied this method to evaluate the creaming behavior of an O/W emulsion [15, 16] and to accomplish real-time measurements of water-in-oil (W/O) emulsions [17] and multiple emulsions [18].

A liposome and an emulsion share a common point of localization with respect to specific components in a microenvironment. We considered that, similar to the previous studies using O/W emulsions, a detectable compound encapsulated into dispersed liposomes appeared as positive spikes in a time profile, which allows the online measurement of liposome-encapsulated compounds without the need of pretreatment. In the present study, the detection of the spike signals arising from a component encapsulated in a liposome and the disappearance of these spikes when liposome collapses were confirmed during online measurement via the use of REMPI–TOFMS.

## Experiment

### Reagents and sample preparation

Lecithin from Egg Yolk (phosphatidylcholine, ca. 70%; lysophospholipid, at more than 99%) and methanol were acquired from Nacalai Tesque (Kyoto, Japan); chloroform, glucose, and sucrose were purchased from FUJIFILM Wako Pure Chemical Industries (Osaka, Japan); all were used for the preparation of liposomes. Triton X-100 (Wako Pure Chemical Industries, critical micelle concentration (CMC): 0.22–0.24 mM) was used to collapse (solubilize) the liposomes. We used 2-phenoxyethanol (the octanol–water partition coefficient log *K*_ow_ = 1.16, Wako Pure Chemical Industries) as a model analyte encapsulated in liposomes. When determining the model analyte, toluene and styrene (FUJIFILM Wako Pure Chemical Industries) were used as a point of comparison. In the present study, a mixture of a cyclohexane (FUJIFILM Wako Pure Chemical Industries) solution containing three compounds was prepared.

There are several methods for the preparation of liposomes [19, 20]; a thin-film hydration method was applied in the present study. In addition, a detectable model analyte was encapsulated by using an analyte aqueous solution when preparing liposomes. The procedure of the preparation of liposomes is as follows. First, 4 mL of a lecithin solution was prepared; the concentration of lecithin was 12.5 g/L of a chloroform–methanol mixture [2:1 (v:v)]. The obtained solution was equally (each 2 mL) divided into two 50-mL vial containers to later prepare a thinner membrane. To prepare each container, the lecithin solution was dried via nitrogen flow and vacuum drying (both for 30 min). Lipid membranes were prepared at the bottom of a vial container. Then, 20 mL of an aqueous mixture of 2-phenoxyethanol (145 mM) and sucrose (100 mM) was added to the vial container containing lipid membranes, and a liposome suspension was obtained via hydration for 2 h. Then, the aqueous solution of an outer phase was replaced with a new aqueous solution without 2-phenoxyethanol as follows. The liposome suspension was centrifuged (4 ℃; 15,000 rpm; 30 min), and 18 mL of the supernatant was discarded. Then, an equal volume of a glucose aqueous solution (245 mM) was added. The same centrifugation was again performed, and a 1.5 mL suspension including precipitated liposomes was taken from the bottom of the vial container. By the above-mentioned procedures, 3 mL (in total) of a liposome sample was obtained, and 1 mL portions of this sample were sequentially passed through an extruder (10,000 nm NanoSizer™ Extruder, T&T Scientific Corporation, CA, United States) with a filter pore size of 10 μm to reduce the particle sizes of the liposomes to less than 10 μm.

### Apparatus

The liposome samples before and after passing through the extruder were observed using an optical microscope (ECLIPSE, TE2000-U, Nikon, Tokyo, Japan) with an objective lens (Plan Fluor, 20 × , NA 0.45 and 60 × , NA 0.70, Nikon) and a digital camera (Digital Sight DS-U1, Nikon).

**Fig. 1:**
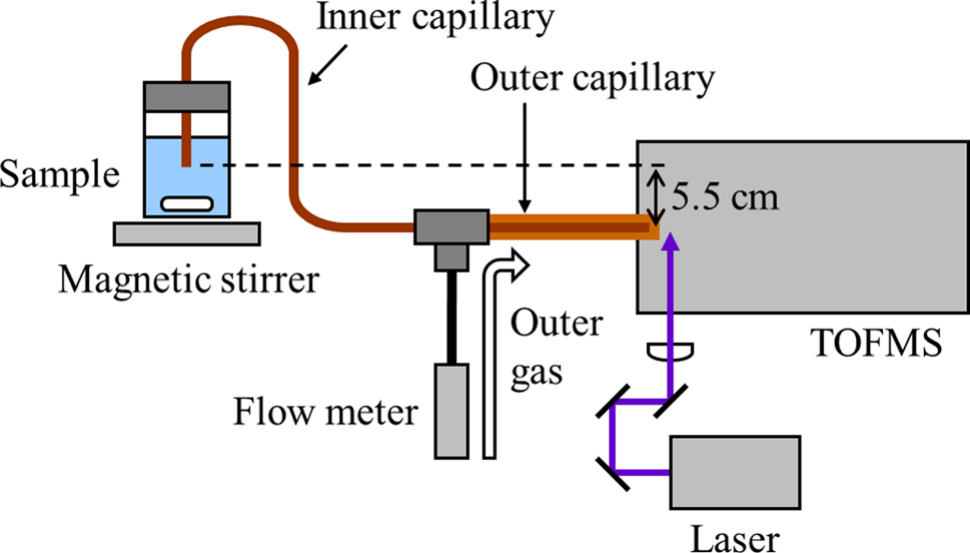
Schematic diagram of the experimental apparatus

A schematic diagram of the REMPI–TOFMS setup used in the present study is shown in Fig. 1. Details of the REMPI–TOFMS are described elsewhere [10], and are only briefly described here. A pair of concentric deactivated fused silica capillary columns (GL Sciences, Tokyo) was used for the sample introduction. Specifications of the inner diameter, the outer diameter, and the length of an inner capillary column are 25 µm, 150 µm, and 70 cm, and those of an outer capillary are 320 µm, 450 µm, and 25 cm. The sample was introduced through the inner capillary column, and ambient air was introduced through the outer version. The flow rate of the ambient air was adjusted to 2 mL/min via a flow meter (RK-1250, Kofloc, Kyoto). The tip of the inner capillary column for sampling was set 5.5 cm above the nozzle for the sample introduction of a mass spectrometer. The fourth harmonic emission of an Nd:YAG laser (GAIA II, wavelength 266 nm, pulsewidth 4 ns, repetition rate 10 Hz, Rayture Systems, Tokyo) was employed as an ionization laser. The pulse energy was adjusted to 20 μJ, and the laser beam was focused via a plano-convex lens with a focal length of 200 mm. The ionization point was adjusted 2 mm away from the tip of the outer column in the mass spectrometer.

Before the measurement of liposomes, 15 mL of an aqueous solution mixture of 2-phenoxyethanol (0.8 mM) and glucose (245 mM) was measured in advance. Then, 3 mL of a prepared liposome sample was added through a gap created by partially opening the lid of the vial container, at which point the recording was simultaneously started using a digitizer (AP240, bandwidth 1 GHz, sampling rate 1 GS/s, Acqiris/Agilent Technologies, Tokyo). Following the addition of a liposome sample, the concentration of 2-phenoxyethanol was calculated to be 3 mM, and 20 min later 2 mL of Triton X-100 aqueous solution (50 mM) was added to the sample, which was calculated to have increased the concentration of Triton X-100 to 5 mM. The sample was gently stirred by a magnetic stirrer (300 rpm) during the measurement. A time profile of the peak areas for 2-phenoxyethanol was constructed by extracting the peak areas of the molecular ion peaks (*m*/*z* 138) obtained from the series of obtained mass spectra.

## Results and discussion

### Research of a model compound for encapsulation into liposomes

First, a model compound for encapsulation into liposomes was explored. As a necessary condition, the compound should be ionized by an ionization laser emitting at 266 nm; typically, most of them are aromatic compounds. In the present study, we considered a hydrophilic compound to some extent to be present in the inner aqueous phase of liposomes. As a result, 2-phenoxyethanol was tested, which was hydrophilic rather than toluene or styrene that we used as analyte compounds for the detection of oil droplets in an O/W emulsion in previous studies [11–16].

**Fig. 2:**
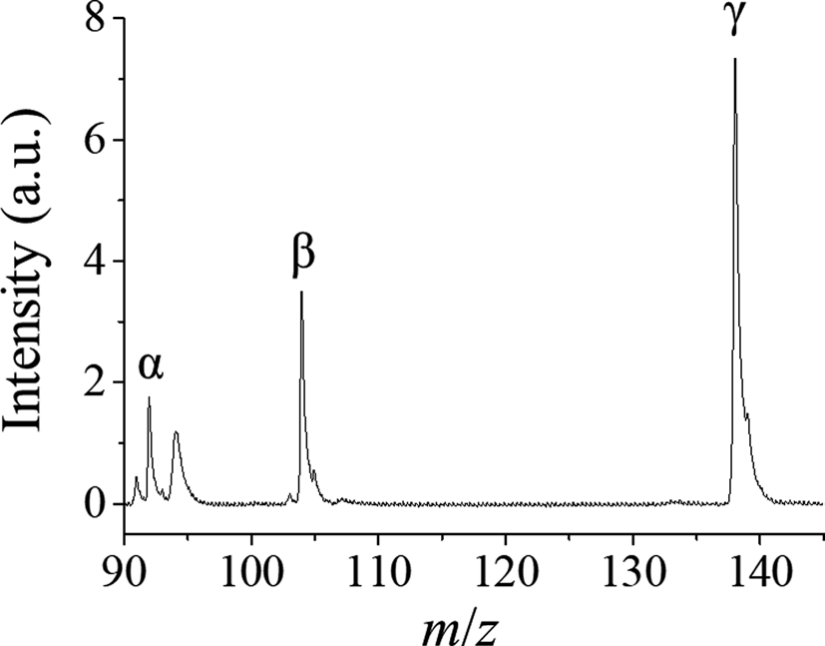
Mass spectrum of the mixture solution. Assignment: α, toluene, *m*/*z* 92; β, styrene, *m*/*z* 104; γ, 2-phenoxyethanol, *m*/*z* 138

The ionization efficiency of these three compounds was first compared. The mass spectrum for the mixture solution is shown in Fig. 2. The molar concentrations of toluene, styrene, and 2-phenoxyethanol were 7.1, 6.4, and 4.9 mM, respectively. The ratio of signal intensity was calculated to be 1: 2.3: 6.3 when comparing equal molar concentration levels. As a result, the ionization efficiency of 2-phenoxyethanol was found to be higher than that of the other compounds under the present experimental conditions, and we decided to use 2-phenoxyethanol as a model compound for encapsulation into liposomes.

### Formation and collapse of liposomes

**Fig. 3:**
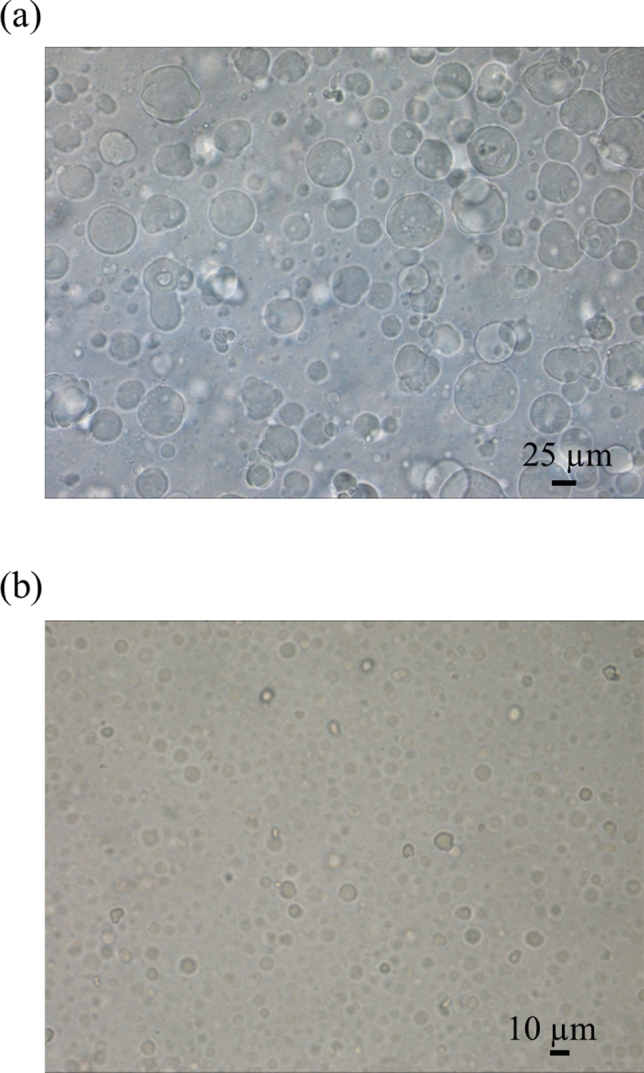
Microscopic images of liposomes before (**a**) and after (**b**) being passed through an extruder

Both before and after passage through an extruder, the prepared liposome samples appeared white and cloudy. Microscopic images of both liposomes are shown in Fig. 3. Before passing through an extruder, many liposomes were larger than the inner diameter of the inner capillary column (25 μm) used for the sample introduction in the present study (Fig. 3a), which resulted in a risk that the capillary column could become clogged when such large liposomes were introduced. Therefore, the use of a filter with a pore size of 10 μm allowed the extrusion process to reduce the size of the liposomes to 10 μm or less (Fig. 3b).

The addition of a surfactant to liposomes to achieve a concentration above the critical micelle concentration (CMC) is reported to cause the disintegration of liposomes, which then should result in the formation of mixed micelles [21, 22]. In the present study, the Triton X-100 aqueous solution was added to the liposome sample to a point at which the concentration of Triton X-100 in the resultant solution was sufficiently higher than its CMC. As a result, the cloudy liquid sample became transparent, and no liposomes could be observed via microscopic observation (data not shown). In this manner, the liposomes were confirmed to truly be disintegrated.

### Real-time measurement of liposomes

Next, the liposome sample containing 2-phenoxyethanol as an analyte species was measured online via REMPI–TOFMS. The anhydrous vesicle bilayer thickness of the egg phosphatidyl choline was reported to be 37 Å [23]. This produced a thickness that was quite small compared with the diameter of the liposomes used in the present study (10 μm or less). Therefore, almost all of the 2-phenoxyethanol analyte species was considered to be in the inner aqueous phase, even when the octanol–water partition coefficient (log *K*_ow_ = 1.16) was considered.

**Fig. 4:**
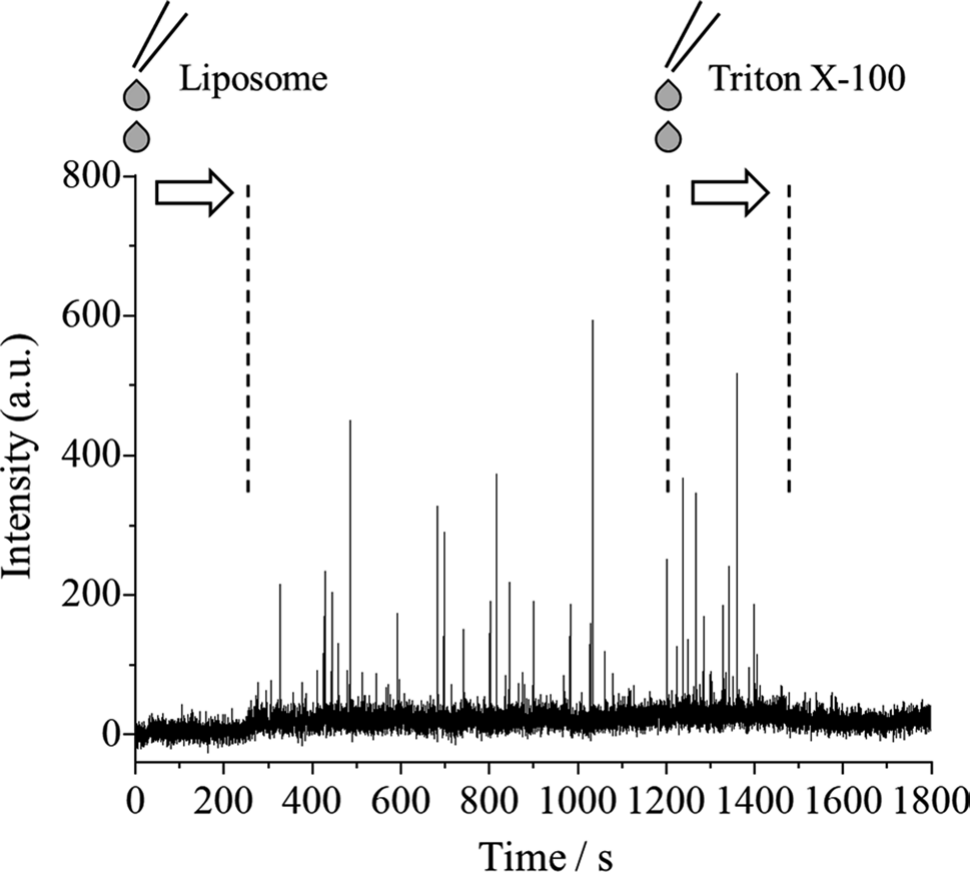
Time profile of the peak areas for 2-phenoxyethanol in a sample. The base signal increased and the spike signals appeared after the addition of a liposome sample, and these spikes disappeared after the addition of a Triton X-100 aqueous solution (see text)

The time profiles of the peak areas for 2-phenoxyethanol appear in Fig. 4. First, an aqueous solution containing 2-phenoxyethanol and glucose (i.e., without containing liposomes) was introduced into TOFMS, and then a liposome sample was added into the 2-phenoxyethanol aqueous solution. The timepoint zero in Fig. 4 refers to the point at which the liposome sample was added. The laser pulses were stopped for the first 30 s to determine the background zero level. After introducing the laser pulses, constant signals appeared, albeit weak. At the point the liposome sample was added into a 2-phenoxyethanol aqueous solution, the liposome-encapsulated 2-phenoxyethanol had not been reached to TOFMS. Therefore, the obtained weak signals are considered to have derived from the 2-phenoxyethanol that had been introduced in advanced to the aqueous solution.

Then, a slight increase in the intensity of the base signal was confirmed ca. 250 s following the addition of the liposome sample. The concentration of 2-phenoxyethanol at an outer aqueous phase was calculated to have increased ca. 4 times following the addition of the liposome sample. Therefore, the obtained signal increase derived from the eventual addition of the liposome sample. The time interval between the addition of the liposome sample and the increase in the base signal (i.e., ca. 250 s) indicated the time required for the sample introduction to TOFMS; the linear velocity of the sample was calculated to be 2.8 mm/s (= 700 mm/250 s). In addition to the increase in the base signal, the spike signals were also confirmed, and were considered to have arisen from the 2-phenoxyethanol encapsulated in the liposomes. That is, while an ionization laser was oscillated at 10 Hz, liposomes were randomly introduced into a TOFMS, and only when highly concentrated 2-phenoxyethanol was ionized via REMPI, spike signals were randomly confirmed.

The signal intensity of spike signals that appear in Fig. 4 was diverse. One of the reasons was that there were liposomes of various sizes under a diameter of 10 µm. Herein, the minimum diameter of a liposome (*D*_min_) that could flow through a capillary column and cause a detectable spike in the time profile was deduced, using the same approach in the previous report wherein the minimum diameter of an oil droplet in an O/W emulsion was calculated [12]. Details are omitted, but the summary is as follows. The spike from the maximum signal intensity (*S*_max_) arises from 2-phenoxyethanol encapsulated into a liposome with a diameter of 10 µm. From the result of Fig. 4, *S*_max_ = 520 was calculated from the average of the signal intensities of the three largest spikes. In addition, the background fluctuation (*B*) was calculated to be 19, and a spike with *S*/*B* = 3 was assumed to be defined as detectable. As a result, the minimum diameter of a liposome, *D*_min_, was calculated to be 3 µm. In this manner, the present method would be useful for the online measurement of compounds encapsulated into relatively large liposomes, such as giant vesicles.

To confirm whether the spike signals of 2-phenoxyethanol were derived from the encapsulated species, a Triton X-100 aqueous solution was added to the liposome sample being measured—that is, the liposomes eventually disintegrated during the measurement process. As a result, the spike signals disappeared following the addition of the Triton X-100 aqueous solution. These results indicated that the spike signals derived from detection of the 2-phenoxyethanol encapsulated in the liposomes.

In the present study, REMPI–TOFMS was applied to the direct analysis of liposomes. Spike signals derived from the liposome-encapsulated 2-phenoxyethanol appeared, and these spikes then disappeared upon the collapse of the liposomes. TOFMS is a non-scanning type of MS, which has enabled us to detect all of the induced ions. Though only one model compound was used in the present study, all detectable analytes can be measured respectively even when several types of analytes are present in a sample. Actually, in the case of an O/W emulsion containing three different analytes, we mentioned the phases in which each analyte existed in the previous report [13]. Similarly, liposomes would be evaluated whether each analyte is encapsulated into the liposomes or exists in an outer aqueous phase. In addition, the spike signals would gradually decrease during the sustained release of an encapsulated compound, which might offer the release rate. Incidentally, we have reported that, using an O/W emulsion, there is a correlation between the sizes of oil droplets and the intensities of spike signals [14]. Therefore, even in the case of liposomes, there is a possibility to estimate the encapsulation efficiency by the combination of the results obtained by REMPI–TOFMS and the size distribution of liposomes. This method selectively detects the liposome-encapsulated compounds without pretreatments, which could be useful for the study of encapsulation efficiency and/or to establish the release rate of liposome-encapsulated compounds.

## Data Availability

The data sets generated and/or analyzed during the current study are available from the corresponding author upon reasonable request.
